# Effect of different cooking methods on sensory quality assessment and in vitro digestibility of sturgeon steak

**DOI:** 10.1002/fsn3.1483

**Published:** 2020-03-05

**Authors:** Qiufeng Feng, Suisui Jiang, Xiao Feng, Xiaodong Zhou, Haiyan Wang, Yujin Li, Jinmei Wang, Shuwei Tang, Yiping Chen, Yuanhui Zhao

**Affiliations:** ^1^ College of Food Science and Engineering Ocean University of China Qingdao China; ^2^ College of Food and Bioengineering Henan University of Science and Technology Luoyang China; ^3^ Hisense (Shandong) Refrigerator Co.Ltd Qingdao China; ^4^ Rongcheng Taixiang Food Products Co.Ltd Rongcheng China; ^5^ Ministry of Agriculture Key Laboratory of Processing of Frozen Prepared Marine Foods Rongcheng China

**Keywords:** cooking method, fish steak, in vitro digestibility, quality acceptability, sous vide, sturgeon

## Abstract

Sous vide can keep the nutritional properties and improve taste of food compared with other conventional methods. In addition, this method may reduce the risk of recontamination after cooking and during storage. The purpose of this paper was to study the effects of four cooking methods (steaming, microwaving, baking, and frying) on the sensory and digestibility on sturgeon steak pretreated by sous vide during the cold storage (0–25 days). The results showed that the digestibility of steaming and microwaving groups (range from 80.34% to 90.12%) significantly higher than that of the other treatment groups (*p* < .05); however, the overall acceptability of the two groups was lower. What more, the frying group has the highest acceptability and the lowest digestibility (range from 65.12% to 70.89%). The springiness (4.12–6.56 mm) and chewiness (1.75–3.12 mm) of the frying group were significantly higher than those of the other treatment groups, which was consistent with the results of scanning electron microscopy (*SEM*) that frying treatment group has a denser structure and smaller pores. With the prolonged refrigeration time, especially between 15 and 25 days, the volatile flavor components (nitrogen oxide, methane, and alcohol) and stagnant water (T_21_) were significantly decreased. Principal component analysis showed that the moisture content was the main factor affecting the overall acceptability and best consumption time of the sturgeon was within 15 days. Simulating the effects of home cooking conditions and refrigeration storage time on the quality of sturgeon steak provided a reference for consumers using similar products.

## Graphical abstract

Sous vide can enhance nutrition and improve taste of food; but which cannot completely kill pathogenic microorganisms in food, so subsequent heating was required. Our study simulated the scene of household cooking and explores the influences of different cooking methods (steaming, baking, microwaving, and frying) on the sensory features, nutrition, flavor, and in vitro digestibility of sturgeon steak within 25 days.

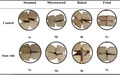

## 1 INTRODUCTION

Sturgeon has high nutritional value including essential long‐chain omega‐3 fatty acids and easily digestible proteins (Hao, Shi, Lai‐Hao, Yang, & Cen, 2008). Furthermore, sturgeon maintains many primitive characteristics such as the lack of intramuscular spines, which can greatly simplify the processing procedure of fish product (Hung, 2017). Caviar is still the most valuable and mature product of sturgeon. However, the high‐value utilization of sturgeon meat was insufficient, which largely restricted the sustainable development of the sturgeon industry (Wang, 2014). Sturgeon steak is one of quick frozen conditioning food, which can expand market of sturgeon and increase the available value of sturgeon meat. Long‐term cryopreservation will make the freezing denaturation of sturgeon steak. Therefore, it is possible to develop the sturgeon processing industry if the quality of sturgeon steak can be maintained and the self‐life can be extended during storage.

Sous vide is an application of a pasteurization thermal process to food products in a hermetically sealed vacuum pouch. It was originally used by French chefs to reduce water loss and shrinkage during cooking for the production of high grade foie gras since the 1970s (Mossel & Struijk, 1991; Ohlsson, 1994; Schellekens, 1996). It allows heat to be efficiently transferred from the water due to vacuum sealing, which can increase the food's shelf life as well as prevent evaporative losses of flavor volatiles and moisture during cooking, leading to enhancement of taste and nutrition (Church & Parsons, 2000; Pino Hernandez, Carvalho, Joele, Araujo, & Lourenco, 2017). Gonzalez‐Fandos et al. have found that sous vide products stored at 4°C showed negligible microbial growth and better acceptable (Nyati, 2000). The results proved that when vacuum‐packed vegetables were stored at 4°C, sous vide combined with microwave heating could preserve some quality, nutritional traits and killed microorganisms effectively of vegetables for up to 30 days (Renna, Gonnella, Candia, Serio, & Baruzzi, 2017). Moreover, it can be concluded that sous vide was the effective way to ensure the safety and extend the shelf life of salmon slices (González‐Fandos, Villarino‐Rodríguez, García‐Linares, García‐Arias, & García‐Fernández, 2005). More importantly, sous vide could improve texture and nutritional properties of meat products. For example, sous vide could enhance the springiness, chewiness and retain moisture of cooked beef steaks compared to controls (Botinestean, Keenan, & Kerry, 2016).

Sous vide has the characteristics of vacuum and low temperature which can effectively improve quality and flavor as shown in Figures S1–S4. Additionally, sous vide can be used to kill microbes effectively. Sensory features, nutrition, flavor, and texture of meat after cooking largely determined consumer satisfaction (Bekhit, Hopkins, Geesink, Bekhit, & Franks, 2014). There is a period of time from producer to consumer through cold chain logistics, and the quality of sturgeon steak will inevitably decline during refrigeration storage. Therefore, it is necessary to explore the effect of storage time and cooking methods on quality of sturgeon steak pretreated with sous vide. Steamed, microwaved, baked, and fried processing generally applied to Chinese home cooking, which can reduce pathogenic bacteria and create sensory.

The objective of this study was to explore the effects of storage time and cooking conditions on sensory features, nutrients, digestibility, flavor, and texture on sturgeon steak pretreated with sous vide. Analyzing the correlations among factors affecting overall acceptability of sturgeon steak treated with sous vide, in order to search a better sturgeon steak cooking method and provide statistics for other researchers. Overall, the findings may help consumers choose the suitable cooking methods and consumption period of sous vide‐pretreated sturgeon steak.

## 2 MATERIALS AND METHODS

### 2.1 Materials and chemicals

Sturgeon (crossbreeds of Russian and Amur sturgeon, 1.5 ± 0.2 kg and 63 ± 8 cm length) was purchased from a local fish market in Qingdao, kept in ice, and transported to the laboratory within 30 min. Oil was obtained from the Walmart supermarket, carrageenan and glutamine transaminase were food grade, and other chemical reagents are analytically pure.

### 2.2 Preparation of the sturgeon steak

#### 2.2.1 Preparation of the sous vide sturgeon steak

Sturgeon steak was prepared as follows. First, the bones, head and skin of the fish were removed to obtain fish meat. Carrageenan (1.5%, w/w) and glutamine transaminase (1%, w/w) were added to sturgeon meat (300 g) and mixed for 3 min. Then, fish meat (100 g) was putted in polypropylene bag and squeeze to form a regular rectangular steak. Thereafter, five groups of vacuum‐packed sturgeon steaks were simultaneously put in 1,000 ml preheated water (65°C）, until the center temperature reached 60°C and heated for 5 min, and then immediately cooled to 10°C. After chilling, the sous vide cooked product was stored at 3 ± 1.0°C central temperature during the cooking process was measured with a digital probe thermometer (NAPUI thermocouple, Mod.TR230X‐8).

#### 2.2.2 Preparation of sturgeon steak using different cooking methods

Sturgeon steaks pretreated with sous vide were cooked by four different cooking methods (steaming, microwave heating, oven‐baking, and frying) after 0, 5, 15, and 25 days. The cooking condition was determined by preliminary experiments. The sturgeon steak was cooked as follows. Steamed: The sturgeon steak was placed in a tray with distilled water at 99.0 ± 1.0°C for 10 min under atmospheric pressure. Microwaved: The sturgeon steak was cooked for 6 min in a microwave oven (WP800S) with a turntable set at 900 W and 2,450 MHz.After 3 min, sturgeon steak was overturned to heat the other side. Baked: The both sides of sturgeon steak were baked for 15 min in a domestic oven (GT12‐01) according to the heating mode: 220°C, hot air, heat up, and down. Samples were turned over every 7 min. Fried: Sturgeon steak was fried in 150 ml of soy oil at 100 ± 5°C in a pan for 6 min. The sturgeon steak was cooked in four methods in triplicate. After heating processing, visible exudates were manually removed from samples and the samples were weighed to obtain the cooked yield and packaged in a plastic bag, and then stored at 3 ± 1.0°C.

### 2.3 Sensory evaluation measurement

The appearance of cooked sturgeon steak varied with the cooking method and storage time as shown in Table 1. The sensory evaluation was carried out by a panel of 10 trained panelists, according to nine‐point hedonic scale (9—Like extremely, 5—Neither Like or Dislike, and 1—Dislike extremely). The samples were divided into four groups depending on four different cooking methods and each group was taken in triplicate followed with the evaluation files and drinking water were provided (Roldán, Antequera, Pérez‐Palacios, & Ruiz, 2014).

**Table 1:** Photos of sturgeon steak cooked with different cooking methods Means in the same column represent different cooking methods and the vertical axis represents different storage times

Days (d)	Cooking methods				
	Control	Steamed	Microwaved	Baked	Fried
0	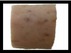	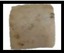	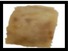	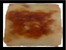	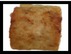
5	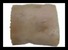	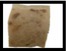	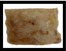	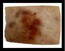	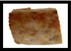
15	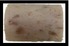	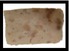	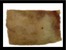	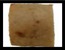	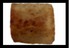
25	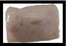	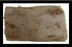	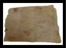	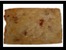	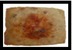

### 2.4 Basic nutrient composition measurement

The moisture, protein, and fat content of cooked sturgeon steak was analyzed by the AOAC official methods (Cunniff, 1995). The results of cooking yield were expressed as the percentage change in the sample weight after cooking compared to initial raw material.

### 2.5 Nuclear magnetic resonance (L‐NMR) measurement

The water state of cooked samples was determined using a Benchtop Pulsed L‐NMR Analyzer P001 (Niumag Electric Corporation) according to the methods of Álvarez and Barbut (Álvarez & Barbut, 2013). Winmuster software can be applied to measure and analyze the relaxation time of low field NMR. Approximately 1.5 g of sample was placed in a glass tube with the diameter of 15 mm and put in the L‐NMR apparatus. The spine‐spin relaxation (T_2_) was measured based on the CPMG sequence. the parameter settings were as follows: τ‐value (200 μs), the proton frequency (22.6 MHz), and the measurement temperature (25°C) (Zhang, Zhang, & Wang, 2015).

### 2.6 Lipid oxidation measurement

Thiobarbituric acid reactive substances (TBARS) were measured according to the methods described by Tavares et al. (2018). The absorbance of the resulting solution was measured at 532 nm with an UV‐Vis spectrophotometer (UV‐2550).

### 2.7 Electronic nose measurement

A PEN3 electronic nose system (Airsense) was used for the acquisition and analysis of data and composed of a measuring chamber with 10 sensors. First, 0.5 g sample was weighed and placed in a 20 ml headspace bottle, which was then maintained in the environment at 25°C for 20 min to equilibrate the volatiles in the headspace. The headspace volatiles were pumped into the sensor chamber at a rate of 300 ml/min. The measurement time and cleaning time are set to be 70 s and 90 s, respectively, to obtain a stable signal. Each sample was tested for three times (Gong et al., 2017). Principal component analysis (PCA) and loading analysis (LDA) were applied to process and analyze data.

### 2.8 Texture profile analysis (TPA) measurement

For TPA, sturgeon steak was cut into cut into small cubes with a side length of 3cm and analyzed by TMS‐Pro texture analyzer equipped with a flat plunger of 50 mm in diameter (P/50) (Food Technology Co). The parameters were set as follows: compression variable: 50%; the speed of the plunger: 60 mm/s; and the height of the plunger: 25 mm (Roldán et al., 2014).

### 2.9 Differential scanning calorimeter (*SEM*) measurement

The samples were fixed with 3% glutaraldehyde solution according to the method of Zhang et al. Then, the samples were rinsed with distilled water for 1 hr in a series of ethanol solutions of 50%, 70%, 80%, 90%, and 100% (v/v). Dried samples were sprayed with gold particles. The samples were observed with a scanning electron microscope (JEOL JSM‐5800 LV) under an acceleration voltage of 15 KV.

### 2.10 In vitro digestibility measurement

The in vitro digestibility of cooked sturgeon steak was investigated according to the method described by Wen et al. (2015). Briefly, two portions of each sample (1 g each portion), one was only treated with pepsin, and the other one was treated with pepsin followed by trypsin. The degree of digestibility was calculated following the equation:

(1) $$\text{DT}=1-\left(W_{i}/W_{t}\right)\times 100\%$$

where DT corresponds to the digestibility of sample, *W_i_* to the weight of dried insoluble protein, and *W_t_* to the total weight samples before digestion.

### 2.11 Statistical analysis

Statistical analysis was performed through analysis of variance (ANOVA) and Duncan's multiple range tests. The results are expressed as mean ± *SD*, and the significant differences were identified at a level of *p* < .05 (Holman, Fowler, & Hopkins, 2016).

## 3 RESULTS AND DISCUSSION

### 3.1 Sensory analysis

The color, odor, and overall acceptability evaluation of sturgeon steak based on the 9‐point hedonic scale was shown in Table 2. The scores for acceptability of steamed and microwaved samples were, respectively, 5.00–6.25 points and 5.59–6.75 points, which were evaluated as “either like or dislike.” For baked and fried samples, the scores of color were, respectively, 7.34–8.25 points and 7.87–8.10 points evaluated as “like much.” The scores of odor were, respectively, 6.50–7.50 points and 7.40–7.75 points evaluated as “like moderately.” During the storage period, especially in 15–25 days, the overall acceptability of baked samples decreased significantly. However, no significant difference was found among fried ones. Sensory evaluation results indicated that consumers preferred baked and fried sturgeon steak compared with microwaved and steamed samples. These results revealed that cooking methods significantly affect color, odor, and overall acceptability of sturgeon steak due to different ways of heat transfer and time (Fabre et al., 2018). Another possible reason may be related to the intensity of Maillard reaction of different cooking methods can produce different flavor substances such as ketones and aldehydes.

**Table 2:** Color, odor, and acceptability of sturgeon steak treated with sous vide. Means in the same column with different letters are significantly different at *p* < .05 by least significant test. Data were expressed as mean ± *SD* (*n* = 10)

	Days	Cooking methods			
		Steamed	Microwaved	Baked	Fried
Color	0	5.25 ± 1.64^aA^	6.80 ± 0.75^bB^	7.80 ± 0.40^bC^	8.00 ± 1.06^aC^
	5	5.42 ± 1.05^aA^	7.25 ± 1.11^aB^	8.25 ± 0.66^aC^	8.10 ± 0.45^aC^
	15	5.25 ± 1.39^aA^	6.125 ± 1.03^cB^	7.88 ± 0.60^bC^	7.87 ± 0.78^aC^
	25	4.88 ± 0.93^bA^	5.00 ± 1.73^dA^	7.34 ± 0.53^cB^	7.88 ± 0.93^aC^
Odor	0	6.50 ± 0.50^aA^	6.60 ± 0.48^aA^	7.40 ± 0.49^aB^	7.71 ± 0.70^aB^
	5	6.00 ± 0.99^bA^	6.75 ± 1.22^aA^	7.50 ± 0.67^aB^	7.50 ± 0.71^aB^
	15	5.5 ± 1.12^cA^	6.00 ± 1.12^aA^	6.50 ± 0.60^bB^	7.40 ± 0.99^aC^
	25	4.25 ± 1.79^dA^	5.50 ± 1.00^aB^	6.88 ± 0.60^bC^	7.75 ± 0.83^aD^
Acceptability	0	6.25 ± 1.30^aA^	6.75 ± 1.30^aA^	8.12 ± 0.01^aB^	7.86 ± 1.13^aB^
	5	6.11 ± 0.97^aA^	6.25 ± 0.66^aA^	8.25 ± 0.83^aB^	8.00 ± 0.71^aB^
	15	5.25 ± 0.97^bA^	5.75 ± 0.66^bA^	7.87 ± 0.60^bB^	7.85 ± 1.00^aB^
	25	5.00 ± 1.20^bA^	5.59 ± 1.03^bA^	6.50 ± 0.70^cB^	7.75 ± 0.93a^C^

Note

Different lowercase letters indicate significant differences between different storage days. Uppercase letters represent significant differences between different cooked methods (*p* < .05). Structured 9‐points hedonic scale: 9—Like extremely, 8—Like very much, 7—Like moderately, 6—Like slightly, 5—Neither Like or Dislike, 4—Dislike slightly, 3—Dislike moderately, 2—Dislike very much and 1—Dislike extremely.

### 3.2 Basic composition analysis

The basic composition of sturgeon steak processed by different cooking methods was shown in Table 3. In steamed samples, the cooking yield and moisture content were, respectively, 82.42%–85.27% and 63.18%–64.89%. The range of moisture content of the sample decreased from 64.77%‐60.01% to 63.18%‐58.53% from 15 to 25 days, the decline trend of baked samples was slower compared with other treatment groups.The lower cooking yield was observed in microwaved, baked, and fried samples. This illustrated steaming can retain the greatest extent water, whereas the water loss of other groups was larger, which was because dry heat processing more easily leaded to dehydration. Moreover, the protein content in steamed samples ranged from 20.34% to 23.43% because the higher internal temperature can promote higher nutrient concentration in that matrix. The fat content of fried sample ranged from 14.64% to 16.90%, which was significantly higher than that of the samples treated with the other three methods due to the absorption of frying oil (Tavares et al., 2018).

**Table 3:** Cooking yields (%) and compositions of cooked samples (per 100 g of sample). Means in the same column with different letters are significantly different at *p* < .05 by least significant test. Data were expressed as mean ± *SD* (*n* = 10)

	Days (d)	Cooking methods			
		Cooking yield (%)	Moisture (%)	Protein (%)	Fat (%)
Steamed	0	85.27 ± 0.79^aA^	64.89 ± 0.63^aA^	20.34 ± 0.09^cC^	9.41 ± 0.46^bB^
	5	84.58 ± 0.41^bA^	64.00 ± 1.67^aA^	21.35 ± 0.09^bC^	10.57 ± 0.96^aB^
	15	84.08 ± 0.38^bA^	64.77 ± 1.17^aA^	21.14 ± 0.53^bD^	10.24 ± 0.20^aC^
	25	82.42 ± 0.07^cA^	63.18 ± 0.16^bA^	23.43 ± 0.41^aC^	9.74 ± 0.20^bC^
Microwaved	0	81.47 ± 0.68^aB^	61.97 ± 1.21^cC^	24.34 ± 0.12^cA^	9.85 ± 0.50^bC^
	5	78.33 ± 0.28^cB^	64.57 ± 1.15^aA^	24.745 ± 0.12^cA^	9.52 ± 0.17^bC^
	15	79.11 ± 0.48^bB^	62.81 ± 0.71^bB^	25.64 ± 0.29^bA^	10.19 ± 0.50^aC^
	25	79.91 ± 0.38^bAB^	60.57 ± 0.71^dB^	26.43 ± 0.13^aA^	9.85 ± 0.63^bC^
Baked	0	79.17 ± 2.01^aBC^	63.95 ± 1.80^aB^	24.43 ± 0.48^bA^	9.51 ± 0.46^bB^
	5	78.12 ± 0.17^bB^	61.06 ± 1.34^bB^	24.43 ± 0.48^bA^	10.75 ± 0.36^abB^
	15	79.26 ± 1.78^aB^	61.13 ± 0.89^bC^	24.03 ± 0.09^bB^	11.08 ± 0.78^aB^
	25	80.72 ± 1.11^aB^	59.00 ± 1.12^cC^	27.3 ± 0.34^aA^	10.44 ± 0.37^abB^
Fired	0	72.59 ± 1.12^cC^	65.07 ± 1.86^aA^	22.12 ± 0.48^cB^	14.64 ± 0.45^cA^
	5	74.94 ± 0.78^aC^	60.84 ± 1.26^bC^	22.12 ± 0.48^cB^	15.44 ± 0.66^bA^
	15	74.10 ± 0.15^abC^	60.01 ± 1.54^bD^	23.44 ± 0.81^bC^	15.30 ± 0.66^bA^
	25	73.43 ± 1.11^bC^	58.53 ± 1.36^cC^	25.57 ± 0.37^aB^	16.90 ± 1.01^aA^

Note

Different lowercase letters indicate significant differences between different storage days. Uppercase letters represent significant differences between different cooked methods (*p* < .05).

### 3.3 Low Nuclear Magnetic Resonance (L‐NMR) relaxation time analysis

**Figure 1:**
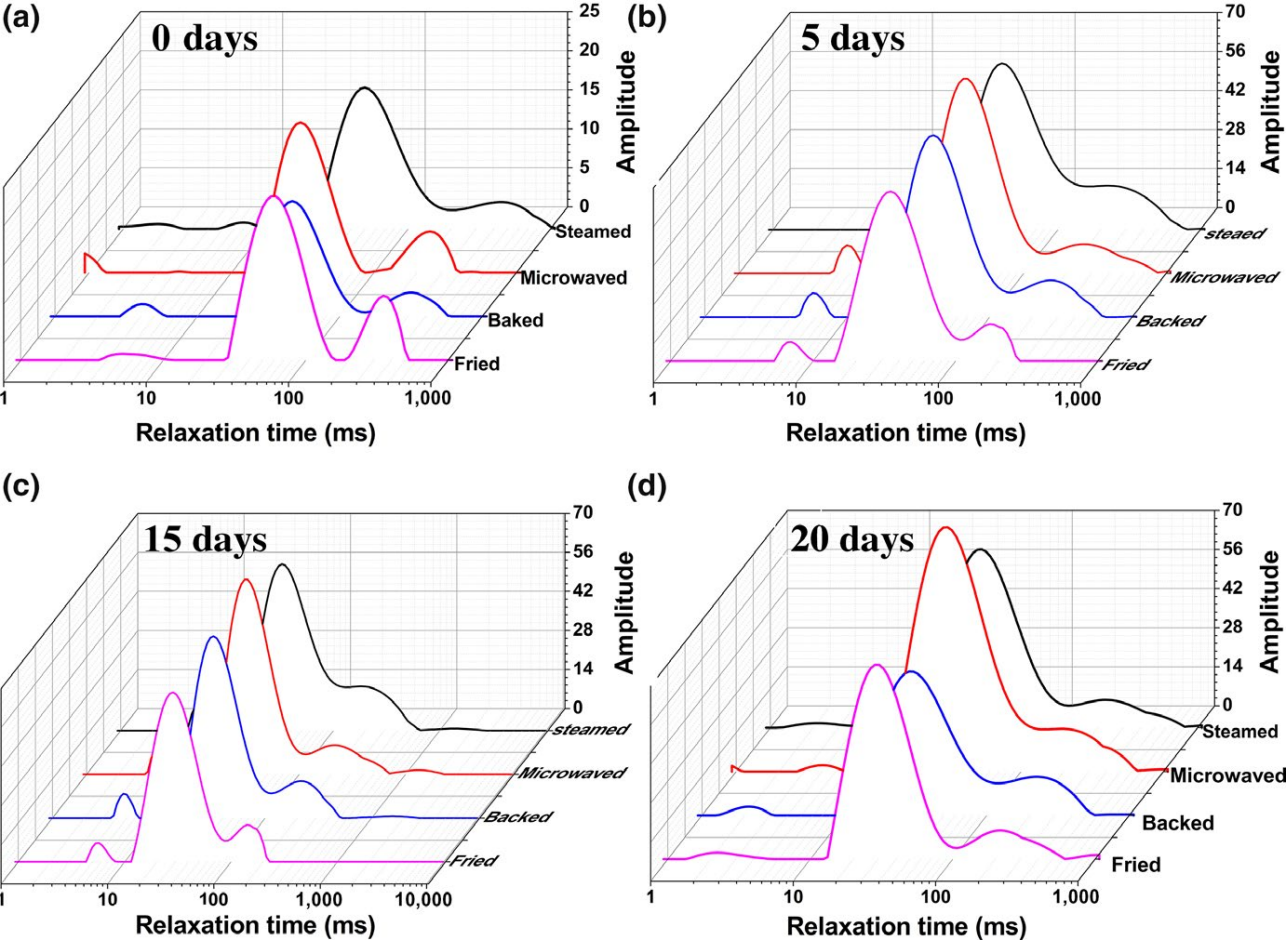
Distribution of T_2_ relaxation times in sturgeon steak treated with four different cooking methods during the shelf life

The transverse relaxation time of L‐NMR can reflect the water state. Additionally, water–macromolecule interactions in food network structure could also affect the water relaxation time (T_21_ and T_22_) (Gianferri, Maioli, Delfini, & Brosio, 2007). As shown in Figure 1, the relaxation curves of different samples exhibited a multi‐exponential behavior due to different structural elements. The peak at T_21_ (1–10 ms) corresponded to the water tightly bound to proteins (Andersen, Andersen, & Bertram, 2007). T_22_ (10–150 ms) corresponded to water molecules trapped within protein network. In addition, the third peak T_23_ (100–500 ms) was ascribed to protons of fat molecules within the steak matrix (Gianferri et al., 2007). As depicted in Figure 1, the relaxation time of all three components was affected by cooking methods and storage time. The fact was probably related to the higher internal temperature and longer cooking time could promote the internal water losses (Tavares et al., 2018). The value of relaxation time shifted to the right with the increase in storage days, indicating that the fluidity of water gradually increased in the later storage period (McDonnell et al., 2013). This tendency was consistent with the change in water content during storage period. Moreover, the trend of relaxation time (T_22_) in fried sturgeon steak moving to the right was less obvious than that in the other groups. This result suggested that there was a least change in the water and lipid behavior of fried samples during storage time, which might be related to the texture and flavor of the fried sturgeon steak.

### 3.4 Fat oxidation analysis

**Figure 2:**
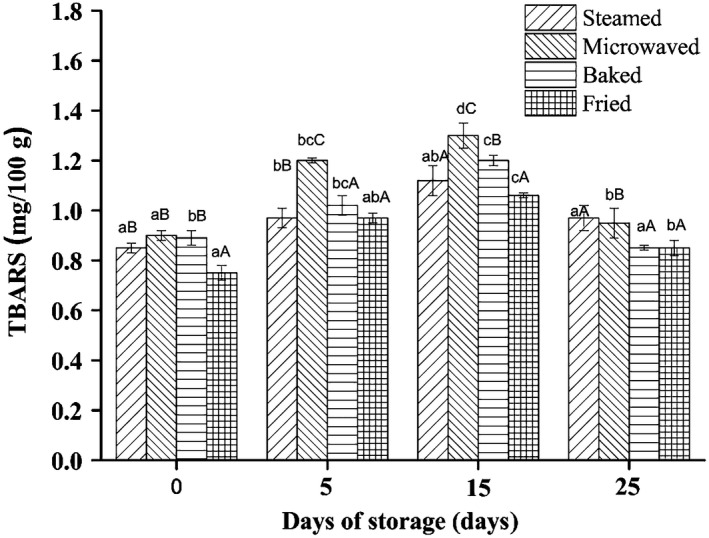
Thiobarbituric acid reactive substances (TBARS) of sturgeon steak treated with four different cooking methods during the shelf life. Different lowercase–uppercase letters indicate significant differences between different storage days. Uppercase letters represent significant differences between different cooked methods (*p* < .05)

Cooking induces lipid oxidation and produces a series of volatile flavor materials, which affect the taste of meat. The secondary oxidation products such as malonaldehyde (MDA) react with protein molecules easily, thus affecting the flavor of meat. The limit of thiobarbituric acid value TBA was set at 2 mg/100g (Connell, 1990). Comparing the changes in the content of MDA in different processing groups, the MDA content of the fried sample was significantly lower than that of baked and microwaved (Figure 2). The reason was that the eventually formed MDA could be lost in the frying oil or formation of compounds with proteins (Narciso‐Gaytán et al., 2011). The other reason for lower MDA content in fried sample may be due to the formation of the Maillard reaction products with dense structure on the surface sample, inhibiting further oxidation inside of sample (Tavares et al., 2018). In general, the results presented no specific trend but values tended rising first and then decreasing, and the highest TBARS values occurred on the 15th days. It may be related to the breakdown of MDA into volatile compounds or to their reaction with protein chains, leading to the formation of Schiff bases (Maqsood & Benjakul, 2010).

### 3.5 Electronic nose analysis

**Figure 3:**
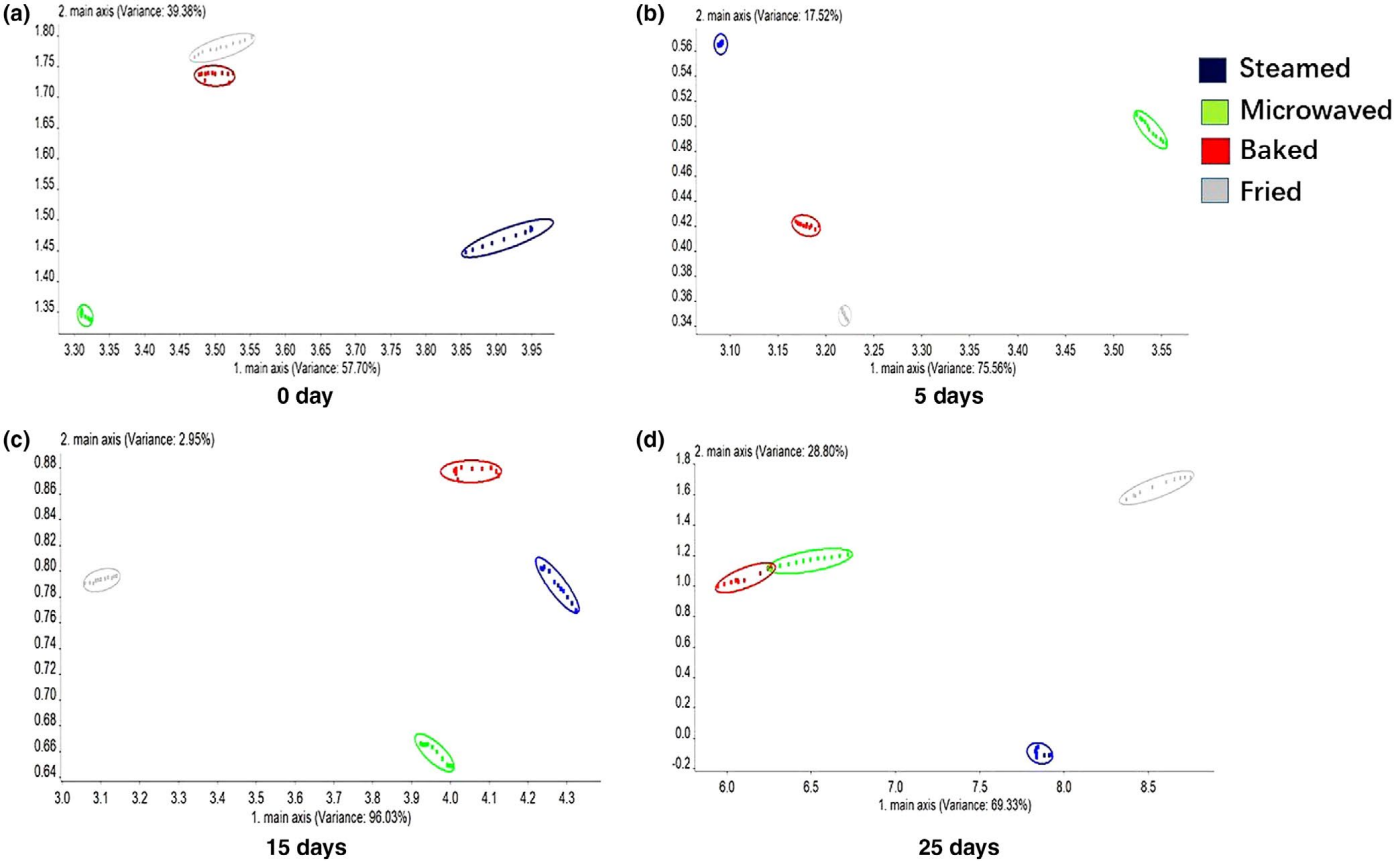
Loading analysis and principal component analysis of electronic nose analysis of sturgeon steak treated with four different cooking methods during the shelf life

The flavor change of sturgeon steak in different cooking methods during storage time was determined by electronic nose that is a device engineered to mimic the olfactory system of humans. The PCA result of sturgeon steak was shown in Figure 3. The sum of the contribution rates of principal components PC1 and PC2 exceeded 95%; therefore, the PC1 and PC2 can represent the overall information. At the early stage of storage, the odor response values of four cooking methods were significantly different. However, the odor response values slightly overlapped on the 25th day, indicating that similar odor generated after storage. The odor of steamed and fried samples was quite different from that of microwaved and baked samples. The possible reason was that the extent of Maillard reaction in four cooked methods was different. Moreover, the odor response values of each treatment group ranged from 0.88 to 3.9 from 15th day to 25th day, indicating great change in odor during this period due to the corruption.

### 3.6 Chewiness, springiness, and gumminess analysis

**Figure 4:**
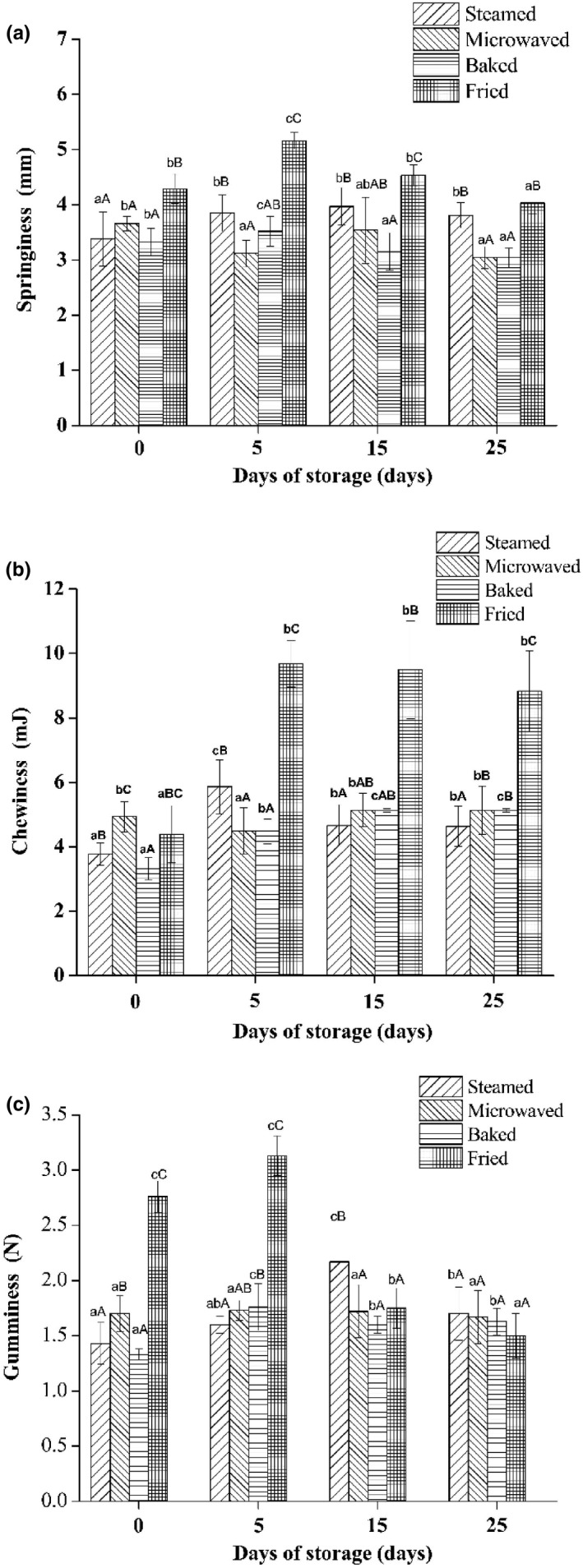
Springiness (a), chewiness (b), and gumminess (c) of sturgeon steak treated with four different cooking methods during the shelf life. Different lowercase letters indicate significant differences between different storage days. Uppercase letters represent significant differences between different cooked methods (*p* < .05)

Texture profile analysis experiments can be used to measure chewiness, springiness, and gumminess for simulating oral chewing (Figure 4). Compared with chewiness and gumminess, there was no significant difference in the springiness of sturgeon steak after four cooking methods. The chewiness of steamed sturgeon steak (range from 3.75 to 4.95 mm) was significantly lower than other treatment groups (*p *＜ .05). The chewiness (range from 4.25 to 9.83 mj) and gumminess (range from 1.75 to 3.21 mm) of the fried sturgeon steak were higher than those of other treatment groups. The reason may be that the formation of a dense Maillard reaction product on the sample surface; therefore, the inside of the sample no longer reacts to form a dense structure. This result confirms with the result of the fried sturgeon steak has less MDA content. But the difference is that a significant difference in 0–5 days for gumminess. The chewiness of fried samples was significantly higher than that of other treatment groups in the 5–15 days. This is consistent with the results of Li et al. (2016) suggesting that long‐duration stewing with prefrying combined with flame heating can improve favor, eating quality, and fat nutritional values, which have potential health benefits in reducing the risk of cardiovascular diseases.

### 3.7 Scanning electron microscopy analysis

**Figure 5:**
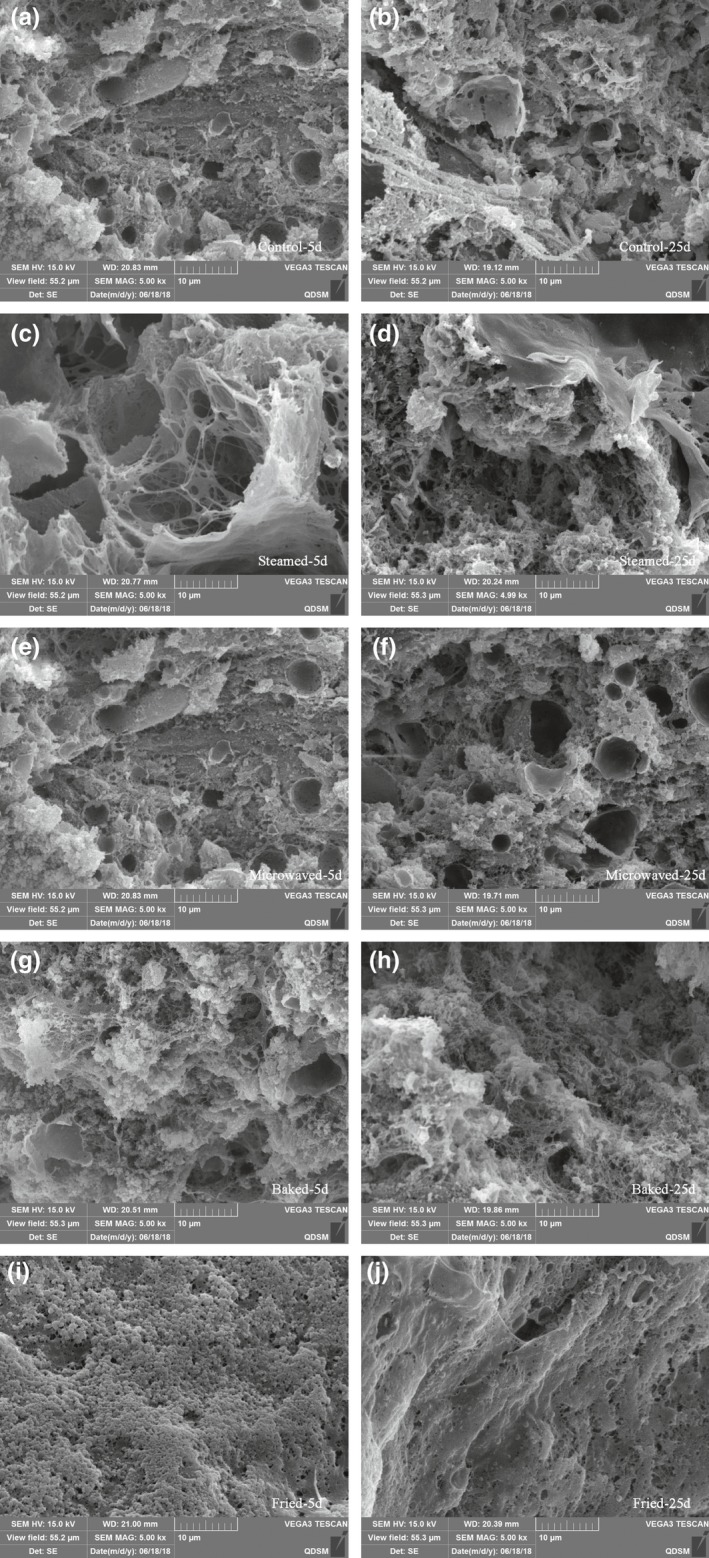
Microstructures (*SEM*, ×5,000) of sturgeon steak treated with four different cooking methods under refrigeration conditions in 5th days and 25th days

Sturgeon steak cooked using different cooking methods were selected to observe microstructure on the 5th and 25th days (Figure 5). The control sturgeon steak exhibited porous microstructures with heterogeneous structure. The sturgeon steak cooked by four different cooking methods also showed porous microstructures with more uniform network structure, indicating that the structure of sturgeon became more compact. This result was in agreement with the reports of Palka and Daun, which the network structure of cooked bovine *M. semitendinosu*s was more compact (Palka & Daun, 1999). Compared with the other cooking methods, the sturgeon steak cooked via frying appeared to have smaller pores embedded in a compact matrix. After 20 days, pore size in these sturgeon steaks had increased and structure becomes rough. Additionally, the microstructures of different samples gradually became loose, indicated that with the extension of storage time, the crosslinking degree of proteins gradually reducing and destroying, but the frying samples were less obvious, in agreement with the TPA results.

### 3.8 In vitro digestibility

**Figure 6:**
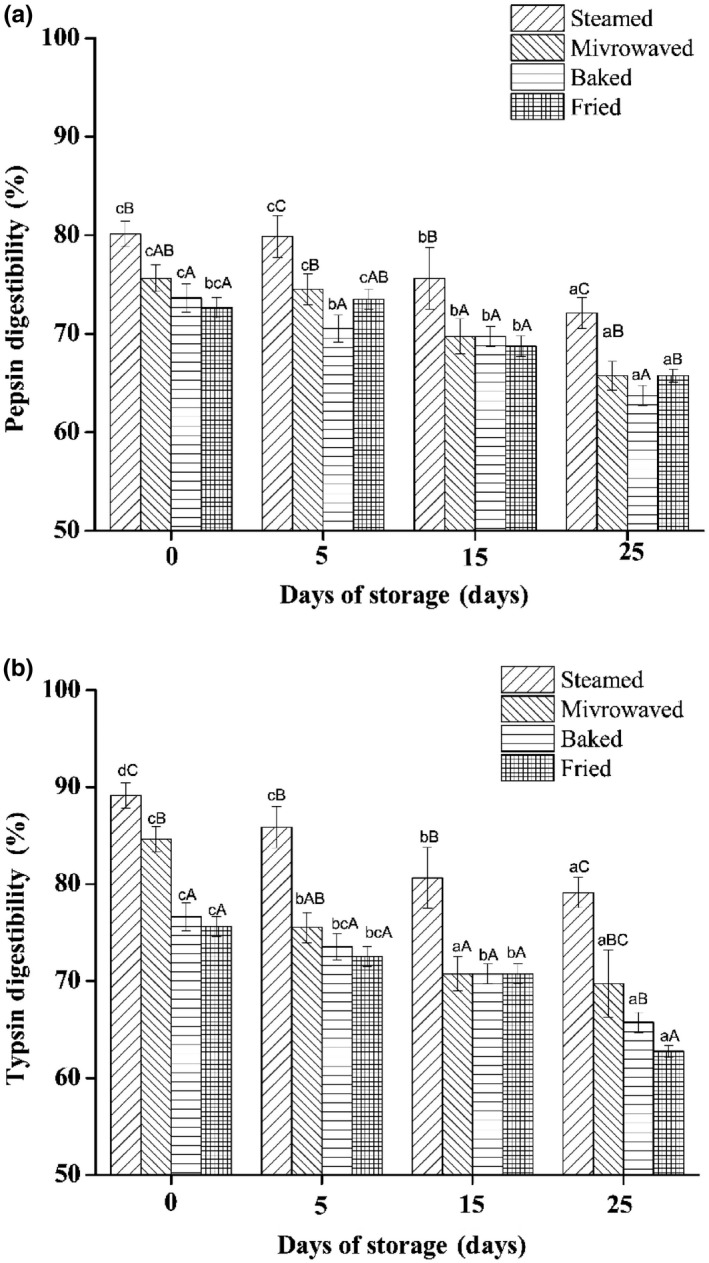
Pepsin (a) and trypsin (b) digestibility of sturgeon steak treated with four different cooking methods during the shelf life. Different lowercase–uppercase letters indicate significant differences between different storage days. Uppercase letters represent significant differences between different cooked methods (*p* < .05)

In vitro digestibility results showed that the sturgeon steak pretreated with sous vide was significantly higher than that of control. Pretreated and cooking methods indicate a significant influence on in vitro digestibility of sturgeon steak treated with pepsin (a) and pepsin & trypsin (b) (*p* < .05). Compared with the control group, the protein digestibility of sous vide treatment groups significantly increased as shown in Figure S3. This may be correlated to the changes in protein conformation and the exposure of hydrophobic sites and cleavage sites in fish protein under low temperature and high pressure environment. The digestibility of pepsin (range from 68.54% to 74.54%) and trypsin (range from 64.54% to 80.78%) in the fried sturgeon steak was, respectively, were lowest (Figure 6.). Moreover, steamed sturgeon steak showed the highest digestibility (range from 74.8% to 88.90%) (*p* < .05). Tavares et al. (2018) also reported that boiled hair tail fillets is easier to digest than fried ones and longer boiling time promoted digestibility (Tavares et al., 2018). These results were due to different lipid, MDA, and Maillard reaction product content. Lipid‐derived aldehydes (like MDA) might interact with protein by carbonyl groups resulted in decreasing the nutritional value of protein. Additionally, the increase in hardness and Maillard reaction product on the surface of baked and fried steak possibly contributed to the decrease of their digestibility (Ferreira, Morcuende, Madruga, Silva, & Estevez, 2018). Furthermore, the proteins were further aggregated and denatured during storage time resulted in the decreasing of digestibility. This hypothesis supported by the digestibility results, the digestibility in four cooking method samples hydrolyzed by pepsin and trypsin significantly decreased with the increase in storage time (*p* < .05).

### 3.9 Principal component analysis

**Figure 7:**
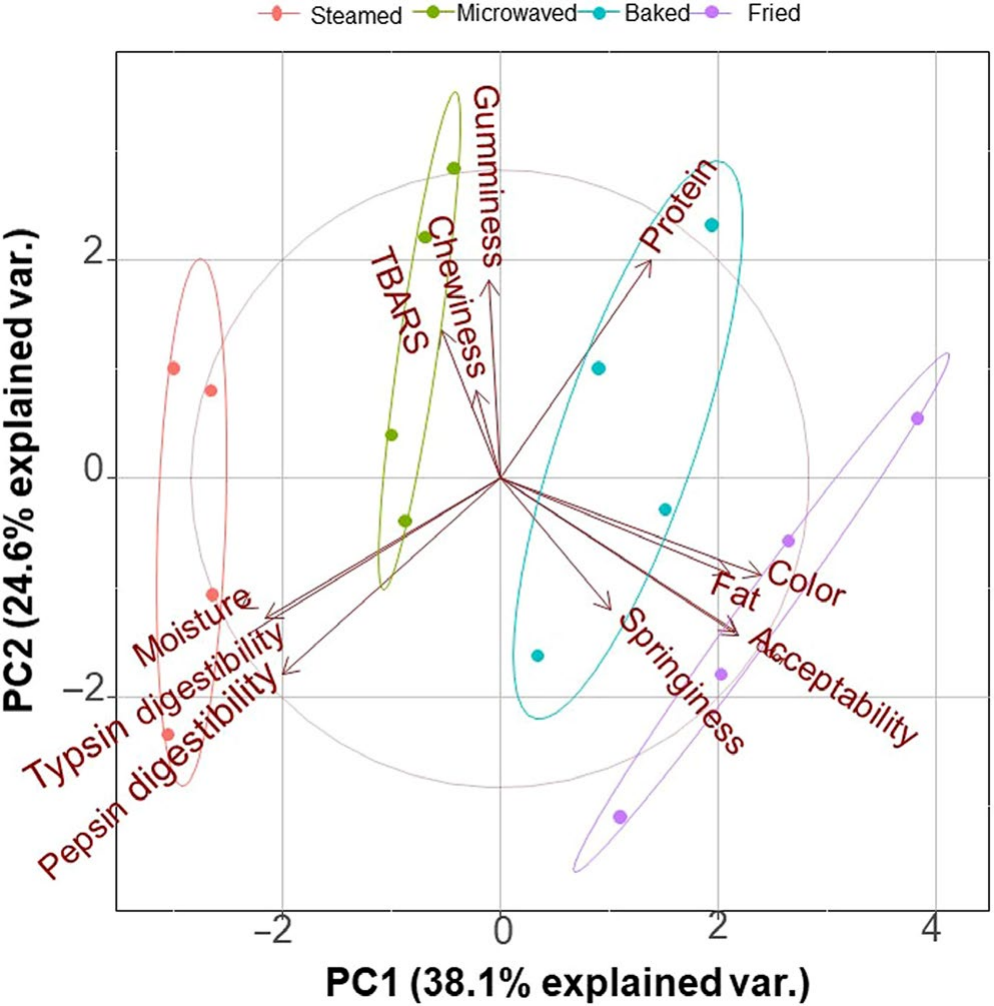
Principal Component analysis the effects of different cooking methods on the quality of sturgeon steak treated with sous vide

The result of principal component analysis showed that moisture content and weight yield were negatively correlated with overall acceptability (Figure 7a). By contrast, springiness, chewiness, gumminess, flavor, and color were positively correlated with overall acceptability. This showed that the change in moisture caused by different heat transfer modes of cooking methods affected the quality of sturgeon steak. Consistent with the results of texture measurement, the springiness, chewiness, and gumminess of fried samples were significantly higher than those of other control groups. The indicators of steamed and microwaved steak were close and slightly overlapped, indicating that the effects of the two cooking methods on the quality of sturgeon steak are smaller. The effect of refrigeration time on the quality of sturgeon steak was studied, which indicated that the quality of the sturgeon steak decreased during refrigeration storage, but did not show obvious regularity (Figure 7b). However, consistent with the results of the previous analysis, the overall acceptability of the fried and baked sturgeon steak is higher. Moreover, the quality of the sturgeon steak declined largely between 15 and 25 days.

## 4 CONCLUSION

Sturgeon steak pretreated with sous vide was cooked in four different ways during the refrigerating storage periods of for 0, 5, 15, and 25 days that, respectively, represent the early, middle, and late stages of the shelf life to simulate the home cooking scene. Fried and baked sturgeon steak with better texture was more easily acceptable and indigestible. The steamed and microwaved sturgeon steak showed higher digestibility than that of other groups. The principal component analysis indicated that the moisture content was an important factor influencing the sensory evaluation, texture, and the overall acceptability. The hardness and chewiness of baked sturgeon steak were lower than other cooking methods, which could appeal for kids and elderly consumers. In addition, the quality reduction was relatively obvious with the prolonged storage time. Based on the basic nutrition, digestibility, and overall acceptability, we will further explore the safety feature of sturgeon steak with different cooking conditions in the future, in order to help choose appropriate cooking conditions.

## CONFLICTS OF INTEREST

There is no conflict of interest in this paper.

## ETHICAL APPROVAL

This study does not involve any human or animal testing.

## Supporting information

FigS1‐S4
