# Supplementary material for: Effect of different cooking methods on sensory quality assessment and in vitro digestibility of sturgeon steak
Source: Food Sci Nutr. 2020 Mar 5;8(4):1957–67. doi: 10.1002/fsn3.1483 (PMC7174215; doi:10.1002/fsn3.1483)
Supplement: Supplementary file 1 — FigS1‐S4 [file FSN3-8-1957-s001.docx]

For convenience, we use the following letters to represent different treatment groups, S for cooking group, M for microwave group, B for baking group, F for frying group, c for control group, and S for sous vide treatment group. The effect of sous vide on the appearance, relaxation times (T_2_), trypsin (a) and pepsin (b) digestibility and SDS-PAGE as follows:

|  | **Steamed** | **Microwaved** | **Baked** | **Fried** |
| --- | --- | --- | --- | --- |
| **Control** | 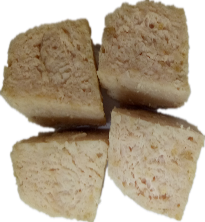  **Sc** | 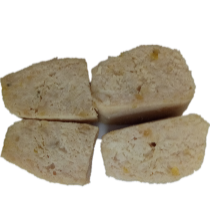  **Mc** | 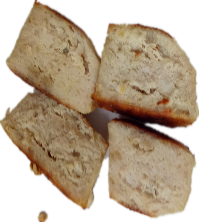  **Bc** | 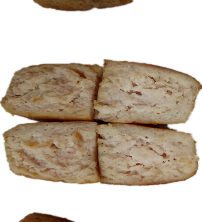  **Fc** |
| **Sous vide** | 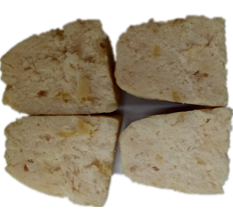  **Ss** | 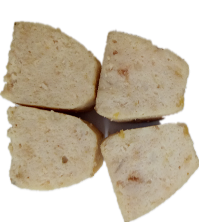  **Ms** | 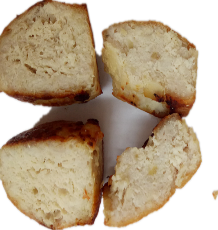  **Bs** | 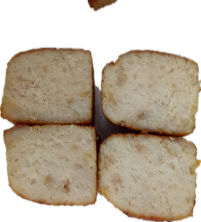  **Fs** |

Figure S1. Appearance of sturgeon fish burger cooked with four different cooking methods.


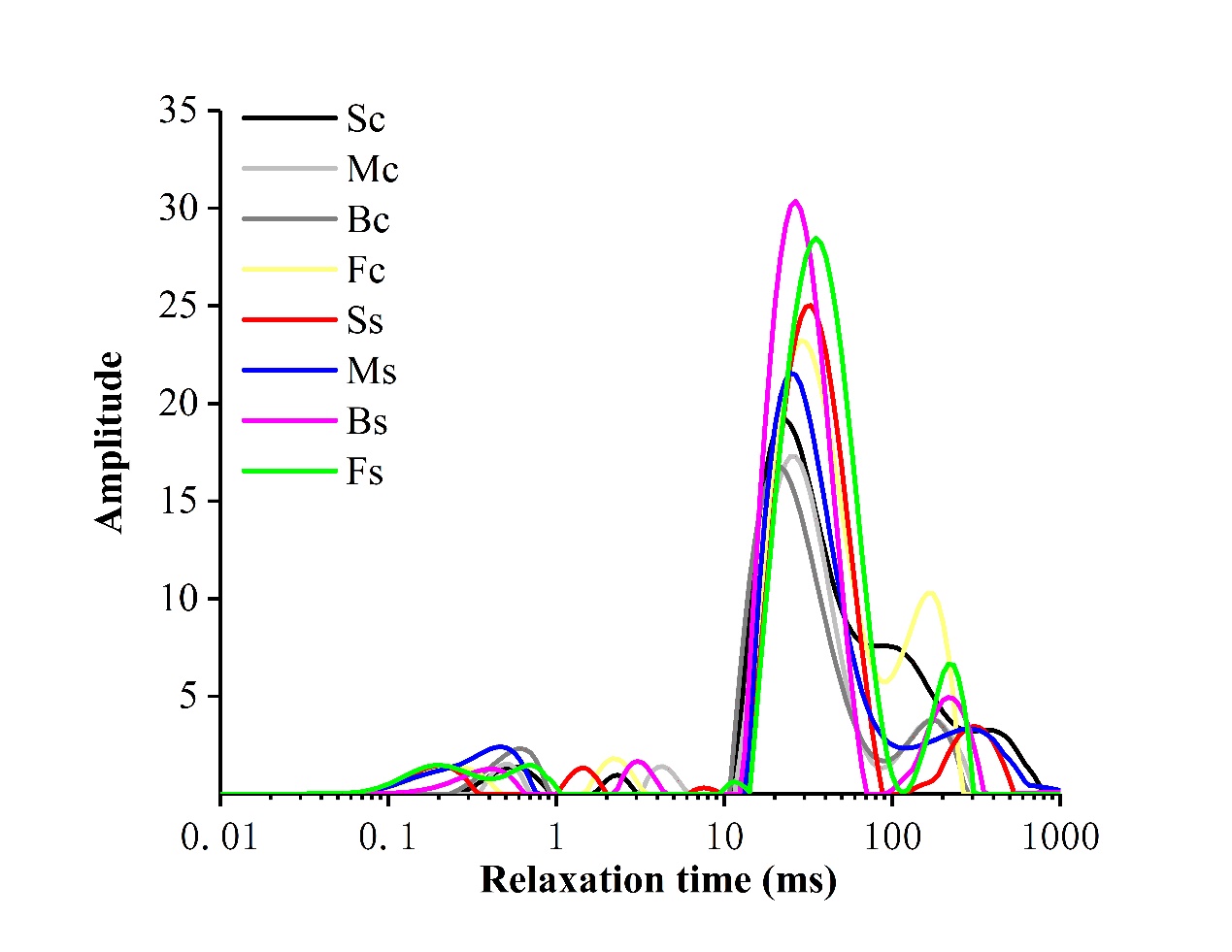


Figure S2. Distribution of relaxation times (T_2_) in surgeon fish burger treated with four different cooking methods.








Figure S3. Trypsin (a) and pepsin (b) digestibility analysis of sous vide on sturgeon fish burger treated with four different cooking methods, *represent *p* < 0.05.


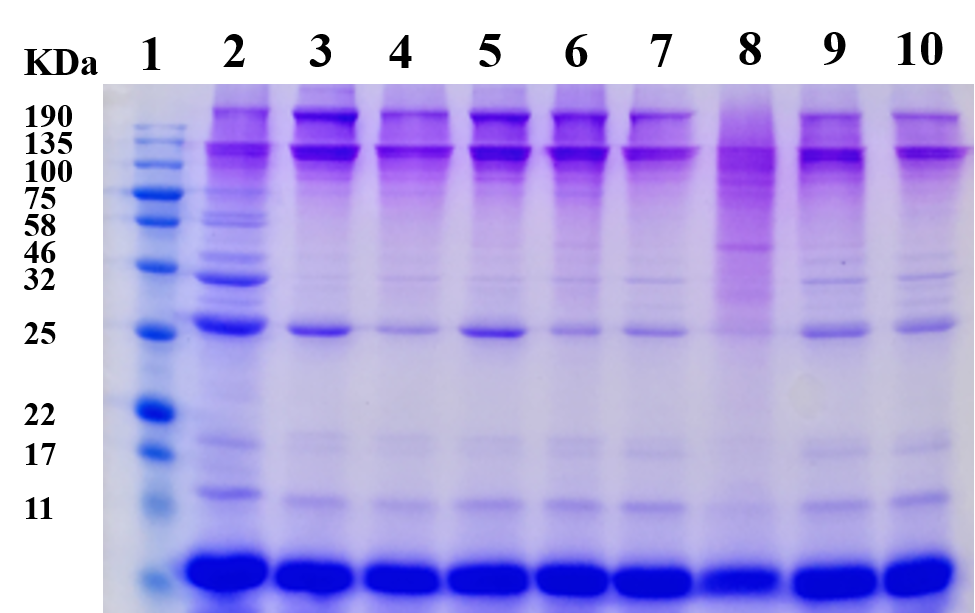


Figure S4. Effect of sous vide on protein denaturation of sturgeon fish burger on SDS-PAGE.
